# Psychological mechanisms of English academic stress and academic burnout: the mediating role of rumination and moderating effect of neuroticism

**DOI:** 10.3389/fpsyg.2024.1309210

**Published:** 2024-01-24

**Authors:** Xiaoyi Zuo, LuLu Zhao, Yue Li, Wanting He, Chengfu Yu, Zhenhai Wang

**Affiliations:** ^1^Laboratory of Philosophy and Social Sciences for Children and Adolescents' Reading and Development, South China Normal University, Guangzhou, China; ^2^Center for Studies of Psychological Application, School of Psychology, South China Normal University, Guangzhou, China; ^3^Guangzhou Laboratory, Guangzhou, China; ^4^College of Teacher Education, South China Normal University, Guangzhou, China; ^5^Journal of South China Normal University, South China Normal University, Guangzhou, China; ^6^Department of Psychology and Research Center of Adolescent Psychology and Behavior, School of Education, Guangzhou University, Guangzhou, China

**Keywords:** English academic stress, academic burnout, rumination, neuroticism, higher education

## Abstract

**Introduction:**

Academic stress is a significant and prevalent phenomenon among college students. According to the Demands-Resources Model, when individuals are unable to cope with stress that exceeds their capacity, burnout may occur. Although English courses hold a significant position in university education, there has been limited research on the mechanisms linking English academic stress to English academic burnout.

**Methods:**

This study recruited 1,130 undergraduate students taking English courses. Participants completed online questionnaires assessing English academic stress, rumination, English academic burnout, and neuroticism traits. A moderated mediation model was constructed to examine the relationship among these variables.

**Results:**

The results indicate that (1) Rumination serves as a mediator in the relationship between English academic stress and burnout; (2) neuroticism significantly moderates the pathway between English academic stress and rumination. Specifically, students with high neuroticism tendencies are more prone to developing rumination when faced with high levels of English academic stress.

**Conclusion:**

These findings offer valuable insights into the psychological mechanisms underlying the association between English learning stress and academic burnout. They emphasize the importance of addressing rumination as a mediator and considering individuals’ levels of neuroticism in interventions aimed at preventing and alleviating academic burnout among university students.

## 1 Introduction

While stress is common at every stage of adolescent development, the number of stressors faced by teenagers increases rapidly after entering college. These stressors include unfamiliar academic courses, adapting to a new school environment, socializing with new peers, and contemplating future career plans. Among the numerous stresses faced by college students, academic stress is particularly significant and prevalent (Hurst et al., 2013). Appropriate academic stress can enhance external motivation for learning in college students. However, excessive stress may negatively impact students’ mental and physical well-being, leading to a range of adverse effects such as academic burnout (Lin and Huang, 2014). Academic burnout refers to a student’s negative attitude and behavior towards studying, manifested as weariness from stress or lack of interest (Salmela-Aro and Upadyaya, 2014). According to the Demands-Resources Model (Demerouti et al., 2001), when individuals are unable to cope with demands that exceed their capacity (such as facing unmanageable academic tasks), burnout may arise.

English holds a significant position among the various courses in university education, as it serves as a compulsory course for nearly all college students in China (Lin and Reinders, 2019). English proficiency is highly valued in Chinese society, as it not only affects students’ ability to pass English language examinations but also determines their attainment of English proficiency certificates, which in turn influences their success in further education, career prospects and other developmental goal (Chang, 2014). This societal emphasis on English proficiency creates expectations and societal pressure on students to excel in English, leading to additional stress and anxiety about their performance.

Additionally, compared to other Chinese-taught courses, learning a second language like English can invoke diverse and distinctive cognitive and emotional processes (Xin et al., 2019). Mastering a new language requires significant effort, including memorization, vocabulary expansion, grammar rules, and pronunciation practice. These cognitive demands, combined with the need to communicate effectively in English, may contribute to increased stress levels among students (Jiang et al., 2021), resulting in a higher prevalence of academic burnout. Thus, it is essential to investigate the relationship between English language learning pressure and academic burnout among the college student population.

### 1.1 The mediating role of rumination

Although the relationship between academic stress and academic burnout has been explored in numerous studies, the underlying mechanisms connecting these two constructs remain unclear (Cheng and Lin, 2023; Gao, 2023). Empirical evidence suggests that academic stress does not directly lead to academic burnout (Hao et al., 2022). Instead, academic burnout arises only when stress is perceived negatively or exceeds an individual’s coping capacity, resulting in a detrimental impact on their mental and physical well-being (Chang et al., 2017; McEown et al., 2023). We propose that a negative cognitive pattern, such as rumination, is likely to serve as a potential mediating mechanism between academic stress and academic burnout.

Rumination, as a negative thinking pattern, often involves repetitively and passively focusing on the causes and consequences of negative states, rather than actively addressing and resolving problems to alleviate those negative states (Nolen-Hoeksema et al., 2008). Control Theory (Carver and Scheier, 1982; Watkins, 2008) provides a direct explanation for how English learning stress leads to rumination: the discrepancy between English learning goals or expectations and current learning achievements creates English learning stress, which compels individuals to constantly contemplate how to reduce this discrepancy. When they are unable to change or control the situation, individuals repetitively dwell on the psychological distress caused by this discrepancy, thus giving rise to rumination (Roberts et al., 2013). Furthermore, stress may induce rumination by undermining individuals’ self-regulation and self-control abilities (Baumeister et al., 2006). When stress surpasses a student’s coping capacity, they may abandon proactive coping strategies (such as problem-solving) to alleviate stress, thereby increasing the likelihood of rumination. Consistent with the aforementioned hypothesis, previous studies consistently found a positive correlation between different sources of stress and rumination (Ye et al., 2020; Gil et al., 2023).

In addition, Resource Allocation Theory (Levens et al., 2009; Connolly et al., 2014) proposes that individuals have limited emotional and cognitive resources. When individuals experience rumination due to high academic stress, the repetitive negative thoughts and beliefs continuously deplete their limited cognitive resources, preventing them from engaging in goal-directed behavior and leading to poor academic performance and academic burnout. Consistent with this theoretical framework, research has explored the relationship between rumination and academic burnout (Garratt-Reed et al., 2018; Vinter et al., 2021). For example, Vinter et al. (2021) investigated the relationship between different cognitive emotion regulation strategies and academic burnout among secondary school students and found that rumination had the strongest association with academic burnout.

In summary, previous theories and research have preliminary revealed the mediating role of rumination between stress and burnout. However, to date, no studies have specifically examined this relationship in the context of English learning. Therefore, this study proposes *Hypothesis 1: Rumination mediates the relationship between English learning stress and academic burnout among college students*.

### 1.2 The moderate role of neuroticism traits

When faced with stress, individuals with different personality traits may exhibit distinct coping mechanisms, leading to diverse developmental outcomes. The field of psychology has proposed numerous theories on personality traits, and the Big Five Personality Model is one widely accepted and influential model (Baranczuk, 2019). Neuroticism is one of the fundamental dimensions of the Big Five Personality traits, measuring an individual’s ability to balance negative emotions and maintain emotional stability (McAdams and Pals, 2006). Individuals with high neuroticism exhibit higher levels of negative emotions, such as anxiety and depression, and are more susceptible to stress (Lahey, 2009). Conversely, lower levels of neuroticism are associated with better emotional regulation abilities (Yang et al., 2020). Research by Suls and Martin (2005) found that individuals with high neuroticism reported a higher frequency of daily hassles, experienced more intense negative emotions, and demonstrated stronger reactions to recurrent negative issues.

Previous studies have established a close association between stress and rumination (Sladek et al., 2020; Gil et al., 2023). However, there is a lack of research on how English learning stress among college students affects rumination based on individual differences, such as personality traits. According to the Vulnerability-Stress Model (Belsky and Pluess, 2009), the interaction between individual vulnerability and environmental risk leads to negative outcomes. Individuals with high neuroticism tend to have heightened sensitivity to stress, which depletes their cognitive resources more rapidly in high academic pressure environments (Yusoff et al., 2021). Simultaneously, academic stress may create a greater perceived discrepancy between learning goals and personal states for individuals with high neuroticism, making them more likely to involuntarily ruminate on negative events and thoughts under stressful circumstances (Roelofs et al., 2008).

From an information processing perspective, there is a positive correlation between high neuroticism and the perception of an uncontrollable and threatening environment. In fact, existing research suggests that neuroticism influences the perception of life events and can lead to impaired cognitive abilities. In certain situations, this can result in a “neurotic cascade effect,” where minor habitual issues are magnified (Goldstein et al., 2020). Neurotic cascades refer to the tendency of highly neurotic individuals to appraise neutral events as harmful or threatening, with subsequent negative effects extending to their own experiences or thoughts (Cross et al., 2023). In this study, individuals with high neuroticism are inclined to intensify the severity of English learning stress and amplify negative images of exam failures. Consequently, they may be more prone to evoke negative experiences within themselves and engage in rumination concerning academic tasks.

**Figure 1:**
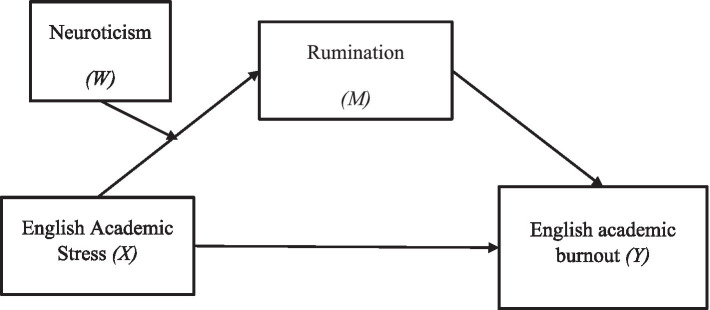
The proposed moderated mediation model.

Thus, we propose *Hypothesis 2: Neurotic personality may positively moderates the relationship between English learning stress and rumination*. The proposed moderated mediation model was presented in Figure 1.

## 2 Methods

### 2.1 Participants

In this study, participants were recruited from university students in multiple universities in Guangdong Province through online surveys. This study only recruited students who were currently enrolled in English courses (i.e., those who had not yet completed their course final exams). A total of 1,203 students completed the survey questionnaire. Samples that did not meet the criteria for questionnaire completion (e.g., having more than 20% missing questions, completion time less than five minutes, or failing more than half of the validity items) were excluded, resulting in a final sample size of 1,130 students. Among them, 406 were male (35.9%) and 724 were female (64.1%). The average age of the participants was 20.27 years with a standard deviation of 1.48, ranging from 17 to 25 years.

### 2.2 Measures

#### 2.2.1 English academic stress

A questionnaire on English learning stress was adapted and revised for this study based on previous relevant scales (Tholen et al., 2022). The questionnaire consisted of four items that asked participants to rate the level of stress they experienced in various aspects of English learning over the past year, such as completing English courses and assignments, taking course tests or proficiency tests. All items were scored on a 5-point scale ranging from 1 (never) to 5 (always). The average score of all items was calculated, with higher scores indicating higher levels of English learning stress. The measurement results showed good internal consistency in this study (Cronbach’s α = 0.91).

#### 2.2.2 Rumination

The assessment of rumination was conducted using four items from the Ruminative Responses Scale developed by Treynor et al. (2003). The questionnaire asked participants to evaluate the frequency of experiencing rumination thoughts when feeling depressed in the past year, such as “I often think about how passive and unmotivated I am.” All items were scored on a 4-point scale ranging from 1 (never) to 4 (always). The average score of all items was calculated, with higher scores indicating higher levels of rumination. The measurement results showed good internal consistency in this study (Cronbach’s α = 0.90).

#### 2.2.3 English academic burnout

The assessment of English academic burnout utilized a modified version of the College Students’ Academic Burnout Scale developed by Lian et al. (2005), consisting of 13 items. The questionnaire asked participants to report their level of burnout in English learning over the past year, with three dimensions including personal achievement (e.g., “I can easily grasp knowledge”), depression (e.g., “I feel exhausted after studying all day”), and maladaptive behavior (e.g., “I rarely study after class”). All items were scored on a 5-point scale ranging from 1 (strongly disagree) to 5 (strongly agree). The average score of all items was calculated, with higher scores indicating higher levels of English academic burnout. The measurement results showed good internal consistency in this study (Cronbach’s *α* = 0.86).

#### 2.2.4 Neuroticism

Neuroticism was measured using the Neuroticism sub-scale of the Chinese Big Five Inventory-15 (Zhang et al., 2019), which consisted of three items (e.g., “I often worry about trivial things”). All items were scored on a 6-point scale, with 1 representing “completely disagree” and 6 representing “completely agree.” The average score of all items was calculated, with higher scores indicating stronger neuroticism tendencies. The measurement results showed good internal consistency in this study (Cronbach’s α = 0.91).

#### 2.2.5 Covariates

Previous research has indicated significant associations between demographic variables such as gender, age, and socioeconomic status (SES) and English learning stress, English academic burnout, and rumination among college students (Gold et al., 2021). Therefore, we included these three variables as control variables in this study. The measurement of family socioeconomic status was assessed using the Social Functioning Rating Scale (Ghaed and Gallo, 2007). This scale presents a ten-level ladder and asks participants to judge their own family’s socioeconomic status. A higher level indicates a higher perceived socioeconomic status of the family. Additionally, participants were asked to report the type of major they were pursuing in university (e.g., humanities and social sciences, natural sciences, engineering and technology), disclose their academic year, provide information about their performance in major courses, and indicate their overall stress levels as covariates.

### 2.3 Research procedure and data processing

This study obtained approval from the ethics committee of the university where the primary author is affiliated. Participating adolescents were required to spend approximately 15 min completing the self-report questionnaire online. They were informed that participation in the survey was voluntary and they could withdraw at any time.

Descriptive statistical analysis of the variables in the study was conducted using SPSS 26.0. Moderated mediation effects were estimated using the SPSS PROCESS 3.5 program developed by Hayes in 2013. Before conducting model testing, all variables were standardized. The significance of regression coefficients in this study was tested using Bootstrapping, a method that evaluates statistical significance based on the standard errors and confidence intervals of parameter estimates. If the confidence interval does not include zero, it indicates statistical significance.

Harman’s single-factor analysis (Aguirre-Urreta and Hu, 2019) was performed on all items of the scales used in this study. Based on the criterion, the maximum factor explaining the variance extracted from all items in this study was 28.33%, which is below the threshold of 40%. Therefore, it is concluded that there is no significant common method bias.

## 3 Results

Table 1 presents the means, standard deviations, and correlation coefficients of the main variables in this study. The results indicate that there is a significant positive correlation among the main variables: academic stress, rumination, and academic burnout. These findings support further examination of the moderated mediation model. The control variables, socioeconomic status, show a significant negative correlation with all four main variables, while age do not have significant effects. Additionally, independent samples *t*-tests were conducted on all major variables to examine the presence of sex differences. The results indicate that significant differences between males and females were observed only in terms of Socioeconomic Status (*t*_(1135)_ = −0.313, *p* < 0.05), with no significant differences found in other variables.

**Table 1:** Descriptive statistics and correlations for the observed variables.

	1	2	3	4	5	5
Age	–					
SES	−0.12***	–				
EAS	−0.03	−0.15***	–			
Rumination	0.01	−0.12***	0.30***	–		
EAB	−0.05	−0.16***	0.39***	0.45***	–	
Neuroticism	−0.04	−0.08**	0.26***	0.54***	0.39***	–
Mean	20.29	2.74	2.99	4.12	2.95	3.43
*SD*	1.06	0.69	1.03	0.68	0.44	1.18

*N* = 1,130; Significant values are shown in bold; SES, social economic status; EAS, English Academic Stress; EAB, English Academic Burnout; *p < 0.05, **p < 0.01, ***p < 0.001.

### 3.1 Test of moderate mediating model

Results of the moderated mediation analyzes are presented in Table 2. This study employed Model 7 from the PROCESS for SPSS proposed by Hayes (2013) to analyze the moderated mediation effect. All variables were standardized. Regression results reveal that in Equation 1, when rumination is the outcome variable, both academic stress (*β* = 0.15, *t* = 5.90, *p*<0.001) and neuroticism (*β* = 0.52, *t* = 20.47, *p*<0.001), as well as their interaction (*β* = 0.05, *t* = 2.03, *p*<0.05), are significantly positively associated with rumination. In Equation 2, when academic burnout is the outcome variable, both academic stress (*β* = 0.24, *t* = 9.16, *p*<0.001) and rumination (*β* = 0.28, *t* = 8.99, *p*<0.001) are significantly positively related to academic burnout.

**Table 2:** Testing the moderated mediation effects of English academic stress on English academic burnout.

	Mode l: Rumination			Mode 2: EAB		
	*β*	*SE*	95% CI	*β*	*SE*	95% CI
CO: Sex	−0.10	−0.05	−0.20, 0.00	0.00	0.05	−0.10, 0.11
CO: Age	−0.01	0.02	−0.06, 0.04	−0.07**	0.02	−0.12, −0.02
CO: SES	−0.07**	0.02	−0.12, 0.02	−0.08**	0.03	−0.13, −0.03
X: EAS	0.15***	0.03	0.10, 0.20	0.24***	0.03	0.19, 0.29
MO: Neuroticism	0.52***	0.03	0.47, 0.57	—	—	—
X × MO	0.13**	0.04	0.06, 0.20	—	—	—
ME: Rumination				0.28***	0.03	0.22, 0.34
*R* ^2^	0.34			0.31		
*F*	96.71***			64.23***		

*N* = 1,130. Sex were coded such that 1 = male, 0 = female. SES, social economic status, EAS, English Academic Stress; EAB, English academic burnout; *p < 0.05, **p < 0.01, ***p < 0.001.CO, control variable; X, independent variable; MO, moderator; X × MO, interaction between independent variable and moderator; ME, mediator.

In terms of demographic variables, socioeconomic status has a significant negative effect on both Equation 1 (*β* = −0.07, *t* = −2.77, *p*<0.01) and Equation 2 (*β* = −0.08, *t* = −3.23, *p*<0.01), while age only exhibits a negative effect in Equation 2 (*β =* −0.07, *t* = −2.73, *p*<0.01). The R2 values for Equations 1 and 2 are.34 and.31, respectively, indicating that the two equations explain 34 and 31% of the total variance in the outcome variables. Both *F*-values are significant, suggesting that both equations are acceptable.

**Figure 2:**
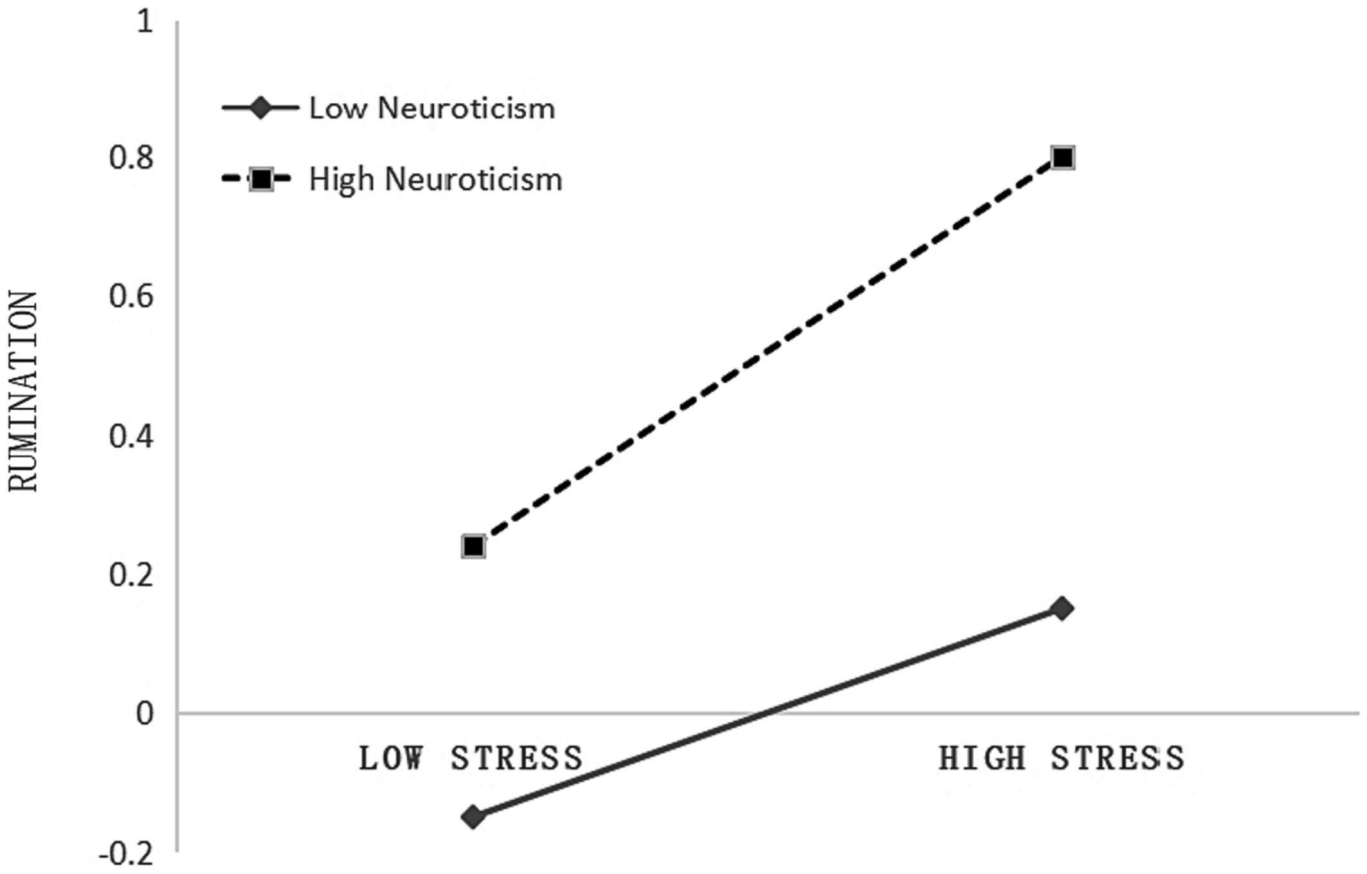
Rumination as a function of English academic stress and neuroticism. Functions are graphed based on two levels of English academic stress: 1 standard deviation above the mean (i.e., High Stress) and 1 standard deviation below the mean (i.e., Low Stress).

To gain a clearer understanding of the interaction effect between academic stress and neuroticism on rumination, simple slope analyzes were conducted. The effects of academic stress on rumination were calculated and plotted using a slope graph when neuroticism was one standard deviation above and below the mean (Mean ± SD). The results (see Figure 2) show the following: (1) In university students with low levels of neuroticism (Mean−SD), there is a significant association between academic stress and rumination (*b_simple_* = 0.21, *t* = 5.78, *p* <0.001, 95% CI = [0.14, 0.28]) and (2) In university students with high levels of neuroticism (Mean + SD), the association between academic stress and rumination (*b_simple_* = 0.27, *t* = 7.92, *p* <0.001, 95% CI = [0.21, 0.34]) is higher compared to the group with low levels of neuroticism.

Furthermore, significance tests using the bias-corrected bootstrap method revealed: (1) In university students with low levels of neuroticism, the indirect effect is significant (Indirect effect = 0.03, *SE* = 0.01, 95% CI [0.01, 0.05]) and (2) In university students with high levels of neuroticism, the indirect effect is higher compared to those with low levels of neuroticism (Indirect effect = 0.05, *SE* = 0.05, 95% CI [0.03, 0.08]).

## 4 Discussion

This study examined the potential mediating role of rumination between English learning stress and academic burnout, based on control theory and resource allocation theory. It also investigated the moderating effect of neuroticism on these relationships, based on the trait Vulnerability-Stress Model. Several meaningful findings were obtained.

Firstly, the results supported a positive correlation between English learning stress and academic burnout. Compared to students in other stages, university students face more sources of stress in addition to academic pressure, such as complicated interpersonal relationships, change in life habits and extensive extracurricular activities (Hurst et al., 2013). Therefore, the escalation of academic stress may contributes to the depletion of individuals’ resources. When these stress exceed students’ coping abilities, they may struggle to effectively regulate themselves, thus making them more susceptible to academic burnout. The importance of English learning for university students is undeniable. However, excessive English learning stress can lead students to develop negative perceptions of their coursework and teachers, questioning the purpose and significance of learning English, and even losing their motivation for studying (Liu, 2015). Therefore, for university educators, it is crucial to avoid overwhelming students with excessive academic pressure by appropriately assigning English learning tasks, fostering students’ internal motivation for English learning, and helping them establish realistic learning goals.

Secondly, the study found that rumination partially mediated the relationship between English learning stress and academic burnout. One possible explanation is that when individuals experience high levels of English learning stress, they may engage in repetitive self-reflection and overthinking about their academic performance, leading to a prolonged focus on negative experiences and emotions. This rumination process can deplete cognitive resources and hinder effective problem-solving and adaptive coping strategies (Rosenbaum et al., 2022). This process involves a feedback loop in which academic stress leads to rumination, further exacerbating negative emotions and impairs students’ ability to meet academic demands. Consequently, stress levels further increase, ultimately resulting in academic burnout.

Therefore, English educators in universities need to adopt appropriate educational strategies to break this cycle. For example, they can encourage students to view mistakes as learning opportunities, shifting their fixed mindset and enabling them to better adapt to and cope with academic challenges. Furthermore, school psychologist should provide social and emotional support for students experiencing high academic stress, while also enhancing their problem-solving skills and emotion regulation skills. These measures will help reduce rumination and enable students to better cope with academic stress.

Finally, the study revealed that neuroticism moderated the relationships among English learning stress, rumination, and academic burnout. University students with high levels of neuroticism are more sensitive to academic stress, more prone to rumination, and more likely to experience academic burnout. In contrast, students with low levels of neuroticism are better able to cope with academic stress, regulate themselves, and reduce the impact of rumination on academic burnout. A potential explanation for these findings is rooted in the trait activation theory (Haaland and Christiansen, 2002), which suggests that individuals with high levels of neuroticism have a stronger emotional reactivity to stressors compared to those with low levels of neuroticism. High neuroticism individuals exhibit heightened sensitivity to academic stress, perceiving it as more threatening and overwhelming. Consequently, this perceived stress triggers a cascade of negative emotions and cognitive responses, including increased rumination.

Additionally, individuals with high levels of neuroticism may have a tendency to interpret ambiguous or challenging situations as personal failures or threats (Abbasi, 2016), leading to an intensified focus on negative self-evaluations and perseveration of negative thoughts. This rumination process perpetuates the experience of stress and amplifies its detrimental effects on well-being and academic performance, ultimately increasing the likelihood of academic burnout. Therefore, understanding individual differences and personality traits is important for implementing targeted educational interventions. Educators can provide training in emotion management and stress coping techniques, while encouraging students to seek social support, in order to enhance their ability to cope with academic stress.

### 4.1 Practical implications

In conclusion, this study explored the relationships among English learning stress, rumination, academic burnout, and neuroticism in university students and obtained meaningful results. From an educational standpoint, these findings suggest several important insights.

Firstly, it is crucial to raise awareness among educators about the impact of English learning stress on students’ psychological well-being and academic outcomes. Educators should be attentive to signs of academic burnout and provide appropriate support and intervention strategies to help students manage and cope with stress effectively. This may involve implementing stress management programs, promoting relaxation techniques, and fostering a supportive learning environment that encourages open communication and emotional expression. Educators can foster a supportive and inclusive classroom environment that encourages open communication and peer support (Wu et al., 2021). Building strong teacher-student relationships and promoting a sense of belonging can provide a protective factor against academic stress for all students (Keane et al., 2023).

Secondly, educating students about the concept of rumination and its potential negative consequences can enhance their self-awareness and empower them to adopt more adaptive thinking patterns. By teaching students cognitive-behavioral techniques, such as cognitive reappraisal and mindfulness-based practices (Hawley et al., 2014; Keng et al., 2016), educators can help students break the cycle of rumination and develop healthier cognitive habits. Additionally, teaching students effective problem-solving skills and time management strategies can assist in reducing excessive stress and preventing the onset of burnout. By integrating activities and exercises that promote self-reflection, emotional regulation, and stress management skills, educators can equip students with the necessary psychological resources to navigate the challenges of English learning effectively (Journault et al., 2023). This approach can foster a positive and resilient mindset, enabling students to perceive English learning as a meaningful and rewarding endeavor rather than a source of overwhelming pressure.

In addition, educators should be aware of the influence of individual differences, such as neuroticism, on students’ susceptibility to academic stress and subsequent rumination. By recognizing the unique needs and vulnerabilities of high neuroticism students, educators can tailor their support and intervention strategies accordingly. Providing targeted interventions, such as stress management workshops or counseling services, can help high neuroticism individuals develop adaptive coping mechanisms and regulate their emotional responses to academic stress. Moreover, educational institutions can promote a proactive approach to cultivating high neuroticism students’ emotional resilience and self-regulation skills. By integrating social–emotional learning programs into the curriculum, students can acquire essential skills, such as emotion regulation, problem-solving, and cognitive reappraisal, which can buffer the impact of stress and mitigate the tendency towards rumination (Rosenbaum et al., 2022).

## 5 Limitation and future direction

The present study has several limitations that should be acknowledged. First, the use of an online survey limits the sample to individuals with internet access, potentially excluding those without such access or who choose not to participate in online surveys. In addition, the current sample consisting of university students from a specific region. This may introduce sampling bias and affect the generalizability of the findings to the larger population of university students in other region. Extend the study to include participants from different regions and diverse university student populations can increasing the external validity and generalizability of the research.

Secondly, despite efforts to ensure the accuracy and reliability of the questionnaire, there may still be subjective errors, recall biases, or social desirability biases among respondents. Additionally, self-report measures may be prone to memory biases or under-reporting of sensitive issues (Brener et al., 2003). Future studies can combine qualitative and quantitative research methods, such as interviews and focus group discussions, to gain a deeper understanding of students’ perspectives and experiences.

Thirdly, although we attempted to infer relationships between variables using theoretical and empirical evidence, the use of cross-sectional design only allows for the investigation of associations between variables at a single point in time. This precludes the ability to establish causality or directionality of effects. Future research could employ longitudinal designs that track changes in variables over time, thus enabling causal inference.

## 6 Conclusion

In conclusion, this study utilized a questionnaire survey to investigate the mediating effect of rumination on the relationship between English learning stress and academic burnout, and the moderating role of neuroticism in this process. The results show that rumination significantly mediates the relationship between English learning stress and academic burnout in university students. In addition, neuroticism has a significant moderating effect on this mediating relationship. Specifically, neuroticism moderates the first half of the mediating path, indicating that students with high neuroticism traits are more prone to engaging in rumination when facing English learning stress. These findings provide valuable insights into the understanding of the psychological mechanisms underlying the relationship between English learning stress and academic burnout. They highlight the importance of addressing rumination as a mediator and considering individuals’ levels of neuroticism in interventions aimed at preventing and alleviating academic burnout among university students.

## Data availability statement

The raw data supporting the conclusions of this article will be made available by the authors, without undue reservation.

## Ethics statement

The studies involving humans were approved by ethics committee of Guangzhou university. The studies were conducted in accordance with the local legislation and institutional requirements. The ethics committee/institutional review board waived the requirement of written informed consent for participation from the participants or the participants’ legal guardians/next of kin because minimized risk: the study involved minimal or no risks to participants, both in terms of physical and psychological harm. Therefore, obtaining written informed consent was deemed unnecessary to protect the participants’ well-being. Preserved anonymity and confidentiality: the online survey ensured that participants’ identities remained anonymous and their responses confidential. By eliminating the need for written consent, we further safeguarded the privacy and confidentiality of the participants. Minimal intrusion: the survey was designed to be brief and non-intrusive, allowing participants to complete it at their convenience without significant disruption to their daily activities. Given the limited impact on their time and schedules, written informed consent was considered unnecessary. Voluntary participation: participants were informed at the beginning of the survey that their participation was entirely voluntary. They were given the freedom to withdraw from the study at any point without providing a reason. As such, the absence of written consent did not compromise the voluntary nature of their involvement. Expedited data collection: waiving written informed consent facilitated a more efficient data collection process, enabling a larger sample size within the designated timeframe.

## Author contributions

XZ: Conceptualization, Writing – original draft. LZ: Conceptualization, Writing – original draft. YL: Data curation, Investigation, Writing – review & editing. WH: Writing – review & editing. CY: Data curation, Formal analysis, Investigation, Methodology, Writing – review & editing. ZW: Conceptualization, Supervision, Writing – review & editing.
